# Variation Pattern of the Elastic Modulus of Concrete under Combined Humidity and Heat Conditions

**DOI:** 10.3390/ma16155447

**Published:** 2023-08-03

**Authors:** Ping Li, Yanru Zhang, Shiwei Duan, Ruiyuan Huang, Jiming Gu

**Affiliations:** 1 School of Mechanical Engineering, Anhui University of Technology, Maanshan 243032, China; 2 College of Civil Engineering, Fuzhou University, Fuzhou 350116, China

**Keywords:** concrete elastic modulus, temperature effect, water effect, humidity–heat coupling factor

## Abstract

The coupling effect of moisture content and temperature on the elastic modulus of concrete is experimentally investigated. The elastic modulus of dry concrete exhibits a clear temperature-weakening effect, while the elastic modulus of wet concrete exhibits a water-strengthening effect at room temperature. Under humidity-heat conditions, the elastic modulus of wet concrete declines with the temperature rise. When the temperature is 20 °C, 200 °C, 400 °C, 520 °C, and 620 °C, the humidity–heat coupling factors of the elastic modulus change rate $D\dot{I}F$ with moisture content are 0.08, 0.07, 0.04, 0.01, and −0.03, respectively, and the declining rate increases with the rise of moisture content. The relation between the humidity-heat coupling factor DIF, moisture content, and temperature was established; The equivalent relation between the water-strengthening effect and the temperature-weakening effect of the elastic modulus was obtained. The temperature range of the strengthening effect and “apparent weakening effect” of water stored inside concrete before heating on elastic modulus was determined; The evolutionary mechanism of the competition between the microcrack expansion and healing of concrete under combined humidity and heat conditions was revealed.

## 1. Introduction

The elastic property of a material is a measure of its stiffness. Estimating the elastic modulus of concrete is necessary to determine the strain-induced stress related to the environmental action and calculate the design stress of concrete members under load and the moment and deflection in complex structures. Studies [1,2,3,4,5,6] have shown that the internal moisture content has an important influence on concrete thermodynamic responses. Concrete structures can be used in the nuclear power industry, chimney kiln structures, and some underwater structures such as dams or piers, where concrete structures may be exposed to both high temperature and high humidity environmental conditions. Therefore, it is essential to know the action mechanism of temperature and moisture on the elastic modulus of concrete materials. For homogeneous materials, there is a direct relationship between density and elastic modulus. However, for heterogeneous composites such as concrete, the elastic behavior of concrete materials is determined by each phase’s volume fraction, density, and elastic modulus and the characteristics of the interfacial transition zone. The external environment of concrete work, such as high temperature, humidity, and load, affects the elastic modulus of concrete by affecting the various phase materials (aggregate, cement mortar and interfacial transition zone between them). An experimental study [7] found that although the stress-strain curves of hardened cement mortar and aggregate show obvious elastic deformation characteristics, concrete is not an elastic material, and the strain of concrete specimens under instantaneous load is neither proportional to the applied stress nor can it be fully recovered during unloading. The nonlinear cause of the stress-strain relationship can be explained by the study on the propagation of microcracks in concrete under load [8]. The static elastic modulus of tensile and compressive action of materials is determined by the slope of the stress-strain curve of concrete under uniaxial load. Because of the nonlinearity of the stress-strain curve of concrete, there are three methods to calculate the elastic modulus. Thus three types of elastic modulus are obtained: (1) The tangent modulus is obtained from the slope of the tangent line at any point on the stress-strain curve. (2) The secant modulus is obtained from the slope of the line between the origin and the point corresponding to 40% of the failure stress. (3) Chord modulus is obtained from the slope of the line between two points on the stress-strain curve. Compared with the secant modulus, the chord modulus replaces the origin with the point corresponding to the longitudinal strain of 50 microstrains. It connects the point corresponding to 40% of the failure stress. The baseline is shifted by 50 microstrains to correct for the slight depression that often occurs at the beginning of the stress-strain curve. The static elastic modulus adopted in this paper is the chord modulus. The study of the elasticity of concrete at high temperatures is a key issue in the mechanical properties of concrete under the thermal-mechanical coupling action, which has been paid attention to by many scholars in this field. Existing studies [9,10,11,12,13,14,15,16] show that the elastic modulus of concrete has a thermal weakening effect, and there are many initial micro-cracks in the interior of concrete specimens, especially in the interfacial transition zone, which greatly influences the stiffness and elastic modulus of concrete. Generally, micro-cracks in the interfacial transition zone can propagate without high energy levels. The interfacial transition zone acts as a bridge between the mortar matrix and the coarse aggregate. Even though the stiffness of the mortar and the coarse aggregate is large, the stiffness of the concrete may be attenuated by the failure of the bridge (i.e., the pores and microcracks in the transition zone) to transfer stress. This way, when the concrete is exposed to fire, its elastic modulus declines much faster than its compressive strength due to cracking in the transition zone. According to the experimental results [17], when the temperature is less than 200 °C, the change rate of the normalized elastic modulus with temperature is about 3.0 times that of the normalized compression strength with temperature; when the temperature is greater than 200 °C, the change rate of the normalized elastic modulus with temperature is about 1.32 times that of the normalized compression strength with temperature. Compared with the thermal weakening effect of the elastic modulus, the water effect on the elastic modulus is still controversial. Although most studies show that water has a strengthening effect on the elastic modulus due to its smaller compressibility and greater viscosity than air [18,19,20,21,22,23], some scholars still believe that the influence of water on the elastic modulus is not significant [24,25], and may even go down [26]. The reason for the insignificant influence of water on the elastic modulus or even the weakening effect may be that the initial cracks inside the concrete are too many due to uneven mixing in the process of concrete production or the relatively low environmental humidity in the process of concrete curing. In the process of soaking, many new crack groups are derived from the initial cracks as damaged nuclei, resulting in the low elastic modulus of concrete. Compared with the study on the variation pattern of concrete elastic modulus under a single condition (temperature or water), the study on the variation pattern of concrete elastic modulus under the humidity-heat condition is rarely reported. The quantification of the coupling effect of water and temperature on the elastic modulus and the study of the variation pattern of the elastic modulus of concrete with different moisture content in different temperature ranges have not been reported. In the early stage, we systematically analyzed the influence of temperature and water on the strength of concrete materials. We found that the strength of concrete decreases monotonously when temperature or water acts alone. In contrast, the strength of concrete increases first and then decreases in a parabolic variation pattern under humidity-heat conditions. The relevant analysis can be referred to Li [27].

The influence of temperature and water on the elastic modulus and strength is very different. To delve into the humidity-heat coupling effect and determine the variation pattern of elastic modulus, the experiments of concrete at different temperatures (20 °C, 200 °C, 400 °C, 520 °C and 620 °C) and different moisture contents (W = 0% (stoved in oven), W = 1.81% (natural drying), W = 2.60%, W = 3.40%, and W = 3.74% (full saturation)) were carried out.

The influence of temperature and moisture content on the elastic modulus of concrete was obtained. It was found that the elastic modulus of concrete increased with the increase of moisture content at room temperature (20 °C); The elastic modulus of concrete with different moisture contents declined with the rise of temperature. The higher the moisture content of the specimen before heating, the more significant the decline of the elastic modulus at high temperatures. The corresponding characteristic temperatures of concrete with different moisture content were obtained, and the quantitative equivalent relation of the temperature weakening effect and water strengthening effect on elastic modulus was established when the temperature was lower than the characteristic temperature. The temperature range of the water strengthening effect and the “apparent weakening effect” of the elastic modulus was given, and the humidity-heat coupling pattern of the elastic modulus was determined. The mesoscopic evolution of microporous structures in concrete under the action of humidity-heat coupling was revealed. In addition, we only analyze the influence of water and temperature on the elastic modulus of concrete, while the influence of other factors on the elastic modulus of concrete is not in the scope of this paper.

## 2. Preparation of Specimen

The concrete specimens used in the experiments were cylinders with a diameter of 50 mm and a length of 100 mm. The mix proportions of concrete material are shown in Table 1.

In this experiment, the curing of the concrete method adopted was in accordance with clause 5.2.4 of the “Standard GB/T50081” [28], currently practised in China. After the specimens were fabricated and cured in the standard way for 28 days, they were placed in a dry indoor environment at room temperature for at least three months. The specimens under this condition were considered natural drying concrete specimens whose mass was recorded as M_0_. The natural drying specimens were stoved in the oven at 105 °C and weighed and recorded several times during the stoving process until the mass changed. Then, they were considered wholly stoved specimens, the mass of which was measured as M_d_. The concrete specimens with different moisture content were obtained by soaking the naturally drying concrete at different times. The moisture content W = (the mass of soaked specimen M − the mass of stoved specimen M_d_)/mass of stoved specimen M_d_, i.e., W = (M – M_d_)/M_d_. The longer the soaking time, the higher the moisture content of the specimen. However, after full saturation, the mass of the specimen has no relation to soaking time. The influences of the age, size, and soaking time on the moisture content of the specimen were obtained (see [27]). The moisture content of the natural drying specimen measured at three months of age was about 1.81%, and the fully saturated specimen with φ 50 mm × 100 mm was about 3.74%.

## 3. Experiment and Results

### 3.1. High-Temperature Experiments

In the experiments, the natural drying specimens were stoved in the oven or soaked in water to prepare five groups of specimens with a moisture content of W = 0% (stoved in the oven), W = 1.81% (natural drying), W = 2.60%, W = 3.40% and W = 3.74% (fully saturated), respectively. The different temperatures (room temperature, 200 °C, 400 °C, 520 °C and 620 °C) uniaxial quasi-static compression tests were carried out on a hydraulic servo M810 equipped with a heating chamber. Before the experiment, we measured the time required to maintain a constant temperature in the heating chamber to ensure an even distribution of internal temperature after reaching the target temperature by drilling a hole in the center of the specimen and inserting a thermocouple. The constant temperature time of 200 °C, 400 °C, 520 °C and 620 °C was 60 min, 120 min, 150 min and 175 min, respectively. Before the experiments, remove the concrete specimens soaked in water for different lengths and wipe the water off the surface. To avoid internal water loss, the specimen should be tested immediately after removal from the water. The experiment was repeated 3–5 times at the same temperature for each moisture content. Experimental equipment, heating system, specific experimental process, and fracture morphology of specimens at different temperatures were detailed in Li [27].

### 3.2. Experimental Results

#### 3.2.1. Variation of Elastic Modulus of Natural Drying Concrete at Different Temperatures

Existing research results show that temperature has a significant effect on elastic modulus. The temperature effect factor K_T_ of elastic modulus was defined as the ratio of elastic modulus E_T_ under high temperature and E_0_ under room temperature of natural drying concrete, i.e., K_T_ = E_T_/E_0_. The variation pattern of K_T_ with temperature T represented the temperature effect of elastic modulus.

**Figure 1:**
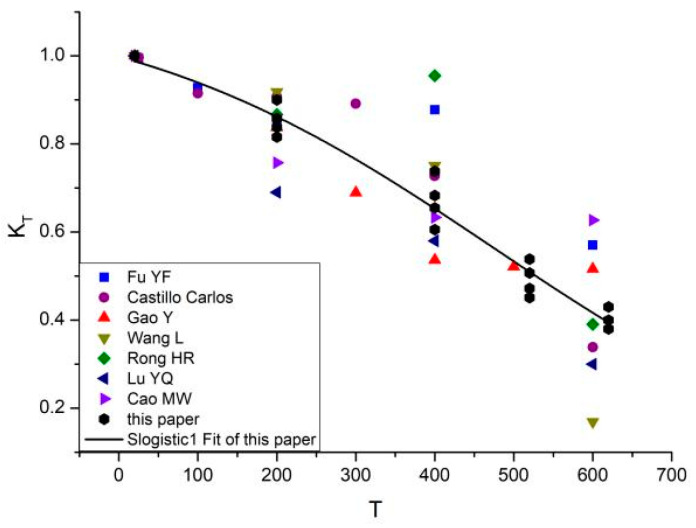
Temperature weakening effect of elastic modulus of natural drying concrete.

Based on the above experiments, the pattern of temperature effect factor K_T_ of natural drying concrete specimen changing with temperature was obtained and compared with previous experimental results, as shown in Figure 1.

It can be seen from Figure 1 that the experimental data in this paper are almost within the normal discrete range of existing experimental results [9,10,11,12,13,14,15], and the elastic modulus of concrete presents a feature of nonlinear monotone decline with the increase of temperature, that is, temperature weakens the elastic modulus. According to the fitting of experimental results, the relation equation of K_T_-T can be written as follows:

(1) $$K_{T}=\frac{a}{1+e^{-{m(T}^{*}-n)}}$$

where T* = T/T_0_, T_0_ is the reference temperature, T_0_ = 20 °C, a = 1.0, m = −0.12, and n = 28.87.

#### 3.2.2. Variation Pattern of Elastic Modulus of Concrete at Room Temperature with Different Moisture Contents

**Figure 2:**
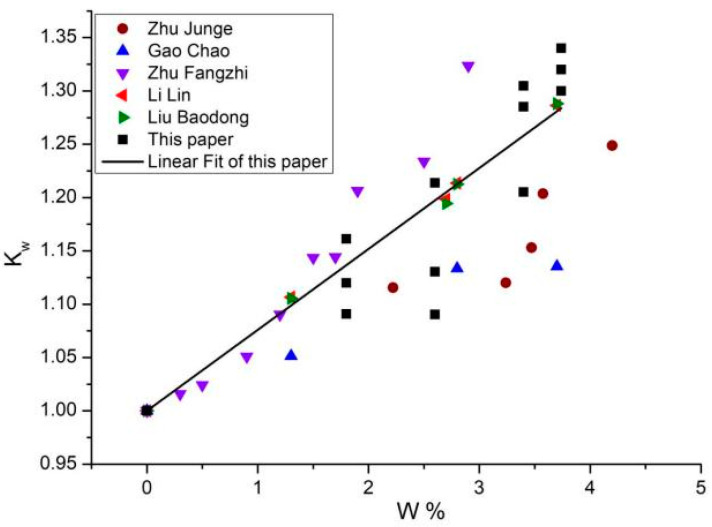
Relation between water effect variation factor K_w_ and moisture content W.

The variation factor that characterizes the effect of moisture content on the elastic modulus of concrete was defined as K_w_ = E_w_/E_d_, where E_w_ is the elastic modulus of the soaked specimen, and E_d_ is the elastic modulus of the stoved specimen. The data of the variation factor K_w_ of elastic modulus with moisture content W was obtained from the above experiments, as shown in Figure 2. It can be found that the experimental results in this paper are consistent with the majority of existing test data [18,19,20,21,22]. The experimental results verify the strengthening effect of water at room temperature on the elastic modulus of concrete.

Based on the experimental data, the relation equation of the water effect variation factor K_w_-W of the elastic modulus was obtained by fitting:

K_w_ = h + gW (2)

where the fitted coefficient h = 1, g = 0.08.

The fitting result shows that the elastic modulus of concrete at room temperature increases linearly with increased moisture content.

#### 3.2.3. Variation Pattern of Elastic Modulus of Concrete under Combined Humidity and Heat Conditions

**Figure 3:**
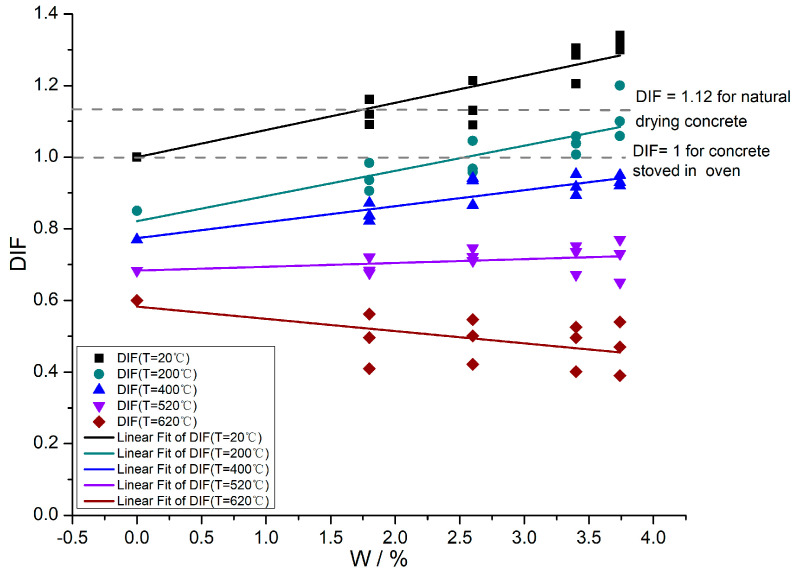
The relationship between the humidity–heat coupling factor DIF and moisture content W.

**Figure 4:**
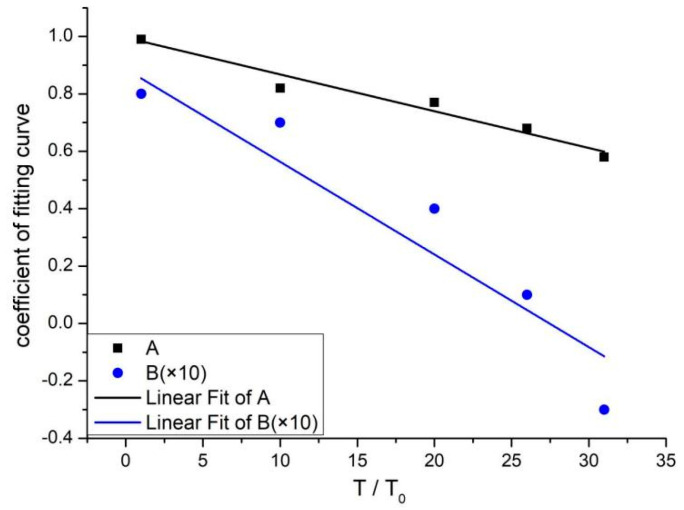
The variation trend of fitting coefficient with temperature.

**Figure 5:**
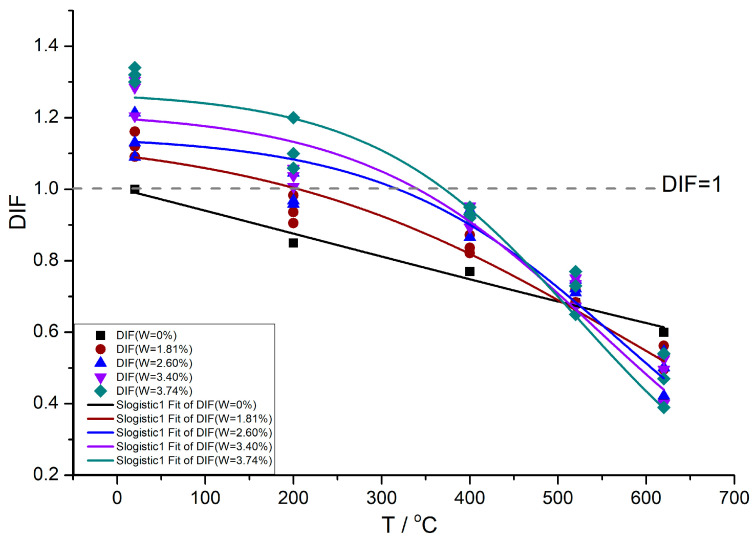
The characteristics of the humidity–heat coupling factor DIF with temperature T.

The high-temperature compression experiment of dry concrete and the room-temperature compression experiment of soaked concrete verified the temperature-weakening effect and the water-strengthening effect of the elastic modulus of concrete. However, under the humidity–heat condition, the concrete water will vanish due to phase changes accompanied by physicochemical reactions. There are very few experimental data on the elastic modulus of concrete under the dual influence of temperature and water, and its variation pattern remains to be determined. Based on the high-temperature experimental result of concrete, the elastic modulus variation factor DIF under the humidity-heat condition was defined as:

DIF = E_Tw_/E_d_ (3)

where E_Tw_ is the elastic modulus of concrete with different moisture content W at temperature T, and E_d_ is the elastic modulus of concrete stoved in the oven at room temperature. To understand the effect of temperature T and moisture content W on the variation factor DIF of elastic modulus, T and W were taken as variables, and the relationship between DIF, T and W was obtained (see Figure 3, Figure 4 and Figure 5). The fitting equation and correlation coefficient are shown in Equations (4)–(10). In engineering practice, Equations (4)–(6) or (7)–(10) can be selected according to specific needs to analyze, design, and predict the elastic modulus of concrete structures under the combined humidity and heat conditions.

The curve equation of the relation between moisture content W and the elastic modulus variation factor DIF under the combined condition of humidity and heat was obtained by fitting:

DIF(T,W) = A(T) + B(T)W(4)

where the parameters A and B are functions of temperature, whose values obtained are listed in Table 2. Their temperature variation is given in Figure 4. (To make the change of A and B can be clearly shown in the same picture, the value of B was enlarged by ten times in Figure 4).

The relationships between A(T) and B(T) with temperature were fitted from the experiment as follows:

A(T) = 1.0 − 0.013(T/T_0_) (5)

B(T) = 0.87 − 0.032(T/T_0_) (6)

where T_0_ = 20 °C.

Equations (4)–(6) reflect the variation of elastic modulus under the humidity-heat condition. By comparing Figure 1 to Figure 4, it can be found that the change of elastic modulus is not the simple superposition of the weakening effect of temperature and the strengthening effect of water, that is, DIF ≠ K_T_K_w_. Their effects on the elastic modulus are coupled with each other. The coupling pattern of the temperature weakening effect and water strengthening effect determines the variation characteristics of elastic modulus. DIF describes the variation pattern of elastic modulus under humidity and heat coupling so that DIF can be regarded as the influence factor of coupling humidity-heat on concrete elastic modulus.

It can be seen from Figure 3 and Figure 4 that:

(1) The elastic modulus of stoved concrete (i.e., W = 0%) decreases monotonically with the increase in temperature, which is the temperature-weakening effect of the elastic modulus. At room temperature (T = 20 °C), DIF(T = 20 °C, W) = K_w_, the humidity-heat coupling factor DIF of elastic modulus increases with the increase of moisture content W, and DIF > 1 are always present at room temperature. The strengthening effect of water on elastic modulus is verified.
(2) Under the combined humidity and heat condition, when DIF = 1, the influence of the water strengthening effect and temperature weakening effect on the elastic modulus is equivalent. Under this condition, the elastic modulus of concrete E_TW_ is equal to that of stoved concrete at T = 20 °C, E_TW_ = E_d_. When T = 200 °C if moisture content W < 2.5%, then DIF < 1 means that the influence of temperature weakening effect on elastic modulus is dominant in the competition between the temperature weakening effect and water strengthening effect. If moisture content W > 2.5%, then DIF > 1 means that the influence of water strengthening effect on elastic modulus is dominant in the competition between water strengthening effect and temperature weakening effect. However, the DIF of T = 200 °C is smaller than that of natural drying specimens at T = 20 °C. When T = 420 °C, 520 °C and 620 °C, the DIF is always smaller than that of the stoved concrete at T = 20 °C.
(3) The elastic modulus of soaked concrete at different temperatures shows different trends. When T = 20 °C, T = 200 °C, T = 400 °C and T = 520 °C, it can be seen that DIF increases with the rise of moisture content W, while when T = 620 °C, DIF declines with the rise of moisture content W. The fitting parameter B(T) describes the change rate of DIF. When B(T) > 0, DIF monotonically increases with the increase of moisture content; when B(T) < 0, DIF declines with the rise of moisture content. As seen from Figure 4, B(T) declines with the temperature rise. That is, the increasing amplitude of DIF gradually declines with the temperature rise. When T = 620 °C and B(T) < 0, DIF no longer increases with the rise of moisture content but shows a decreasing trend.

If B(T) = 0, it can be obtained that the critical temperature of DIF changing with moisture content under the combined humidity and heat condition is about T_c_ = 543 °C (This calculation result is consistent with the experimental fitting result in Figure 5. Considering the fitting curve permissible error, the critical temperature T_c_ of concrete specimens with different moisture content is approximately within the temperature range of 500 °C to 550 °C). When the temperature is higher than the critical temperature T_c_, the influence of the stored water inside the concrete before heating on the elastic modulus of concrete no longer shows a strengthening effect but a “apparent weakening effect”. According to the analysis in Section 4, the “apparent weakening effect” is not directly caused by water, but when the wet concrete is heated to a higher target temperature T (T > T_c_), the water in the concrete has vanished away due to phase changes. A large number of microcracks are distributed inside the concrete. The deterioration of microcracks leads to a low elastic modulus under this target temperature.

The variation pattern of the humidity–heat coupling factor DIF of concrete elastic modulus with temperature T is shown in Figure 5.

According to the fitting of test data, the curve equation of DIF-T is as follows:

(7) $$DIF=\frac{b}{1+e^{{p(T}^{*}-u)}}$$

where, T* = T/T_0_, reference temperature T_0_ = 20 °C, the fitting coefficients b, p and u are only functions of moisture content W, and the values of fitting coefficients under each moisture content are shown in Table 3.

According to Table 3, the variation pattern of fitting coefficients b, p and u with moisture content W can be obtained as follows:

b(W) = 1.73 − 0.48 W + 0.1 W^2^; (8)

P(W) = 0.03 + 0.04 W; (9)

u(W) = 11.03 + 14.51 W − 2.84 W^2^ (10)

The following results can be found in Figure 5:

(1) At T = 20 °C, the higher the moisture content of specimens, the greater their elastic modulus. However, With the increase in temperature, the elastic modulus of concrete with different moisture contents shows a monotonically decreasing trend. And the rate of decrease is related to the moisture content. The higher the moisture content, the faster the high-temperature elastic modulus decreases.
(2) Compared with the elastic modulus of concrete stoved in the oven, when the temperature reached 500 °C, the elastic modulus of concrete with different moisture content is smaller than that of stoved concrete at the same temperature. The DIF(T,W)-T curves of concrete with different moisture content and DIF(T,W = 0%)-T curves of stoved concrete intersect very closely, and almost all of the intersection points are in the temperature range of 500–550 °C. The critical temperature T_c_ = 543 °C calculated by fitting Equation (6) is also in this temperature range. Considering the permissible error of fitting results, the temperature range between 500–550 °C can be considered the critical temperature interval. When the ambient temperature of wet concrete is within the critical temperature interval, the elastic modulus of wet concrete is a little different from that of stoved concrete at the same temperature. The strengthening effect of water on the elastic modulus in micropores offsets part of the weakening effect of crack propagation caused by water phase change on the elastic modulus. The unoffset weakening effect is equivalent to the temperature-weakening effect of stoved concrete. In other words, the comprehensive influence of initial moisture content in concrete within the critical temperature interval on elastic modulus is not apparent. In this critical temperature interval, the elastic modulus of wet concrete is close to that of concrete stoved in the oven. When the temperature T < 500 °C, although the elastic modulus of concrete specimens with higher moisture content decreases faster with the increase of temperature, the elastic modulus of wet concrete is still higher than that of stoved concrete at the same temperature, and the influence of water on the elastic modulus still shows a strengthening effect; When the temperature T > 550 °C, The higher the initial moisture content of concrete specimens before heating, the smaller the elastic modulus with the increase of temperature. The initial water stored inside the concrete before heating decreases the elastic modulus. Whereas, through the analysis in Section 4, it can be seen that the decrease of the elastic modulus in this temperature interval Is not due to the weakening effect of water but the weakening effect of the derived cracks inside the high-temperature concrete after the water occurs phase change and disappears.
(3) When DIF = 1, as mentioned above, the influence of water strengthening and temperature weakening on the elastic modulus of concrete is equivalent, and the two effects can offset each other. That is, the competition between the temperature-weakening effect and the water-strengthening effect resulted in the elastic modulus of the concrete under combined humidity and heat condition being equal to that of the stoved concrete at T = 20 °C. According to the intersection point between the fitting curve DIF-T of concrete under different moisture content and the straight line DIF = 1 (DIF of stoved concrete at T = 20 °C) in Figure 5, it can be concluded that the higher the moisture content is, the more the intersection point moves back. The temperature at the intersection point corresponding to moisture content W is called characteristic temperature T_w_. According to the fitting results, the higher the moisture content, the higher the corresponding characteristic temperature T_w_, which can be referred to in Table 4.

According to Table 3, when DIF = 1, the relation equation between moisture content and its characteristic temperature T_w_ is

(11) $$W=b_{1}+b_{2}e^{-T_{w}^{*}/b_{3}}$$

where, $T_{w}^{*}$ = T_w_/T_0_, T_0_ = 200 °C, b_1_ = −5.81, b_2_ = 5.68, b_3_ = −36.74.

**Figure 6:**
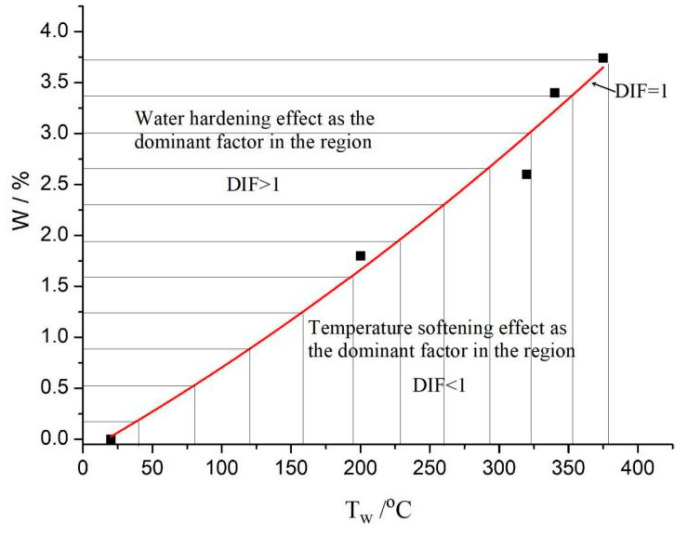
Relationship between moisture content and characteristic temperature T_w_.

When the relation between the moisture content of concrete and the ambient temperature satisfies Equation (11), DIF = 1, that is, the temperature weakening effect and water strengthening effect on the elastic modulus are equivalent and can offset each other; The fitting curve of moisture content W and its corresponding characteristic temperature T_w_ is shown in Figure 6. 

In the region below the curve, the temperature weakening effect is dominant, and the elastic modulus is smaller than that of stoved concrete at T = 20 °C, that is, DIF < 1. The DIF value of the concrete in this region can return to the humidity and heat equilibrium curve (DIF = 1) by increasing the moisture content or decreasing the temperature. On the contrary, the region above the curve is dominated by the water strengthening effect, and the elastic modulus is larger than that of stoved concrete at T = 20 °C, that is, the DIF > 1. The DIF value of the concrete in this region can return to the humidity-heat equilibrium curve (DIF = 1) by increasing the temperature or decreasing the moisture content. Therefore, Equation (11) can also be regarded as the equivalent relation of humidity and heat influence on the elastic modulus of concrete.

#### 3.2.4. Comparison of Elastic Modulus and Compressive Strength of Concrete under Humidity-Heat Condition

**Figure 7:**
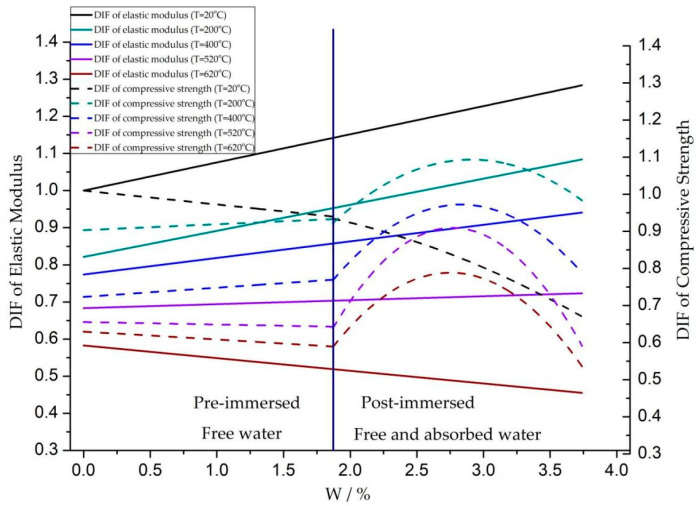
Comparison of elastic modulus and compressive strength with moisture content.

According to the experimental results, the variation of elastic modulus and compressive strength at different temperatures and moisture contents were compared, as shown in Figure 7.

In this figure, the change factor of compressive strength DIF = σ_Tw_/σ_d_, where σ_Tw_ is the compressive strength with different moisture content W at temperature T, and σ_d_ is the compressive strength of concrete stoved in the oven at T = 20 °C. The relevant work on the variation of concrete compressive strength with moisture content before and after water absorption was detailed in [27].

As seen in Figure 7, when the naturally drying concrete was soaked in water, the compressive strength of concrete before and after water absorption showed significant differences with the change of the moisture content. However, no such phenomenon was found when we analyzed the variation pattern of the elastic modulus with the moisture content under the combined heat and moisture condition. That is, there is little difference between free water inside the natural drying concrete and the absorbed water in the soaking process on the elastic modulus, and the influence of two hydration effects on the elastic modulus is not significantly different. It was found that the compressive strength declined monotonically with the rise of moisture content at room temperature, and the elastic modulus showed an opposite trend; Before water absorption, when the temperature was 200 °C and 420 °C, the compressive strength and elastic modulus increased with the rise of moisture content. When the temperature was 520 °C, the compressive strength began to decline with the rise of moisture content, while the elastic modulus increased slightly with the rise of moisture content. When the temperature was 620 °C, the declining trend of compressive strength was more evident with the increase in moisture content, and the elastic modulus also showed an apparent declining trend with the rise of moisture content; After water absorption, at temperatures of 200 °C, 400 °C, 520 °C and 620 °C, the compressive strength first increased and then declined with the rise of moisture content, while the variation pattern of elastic modulus before and after water absorption remained unchanged.

## 4. Mechanism Analysis

**Figure 8:**
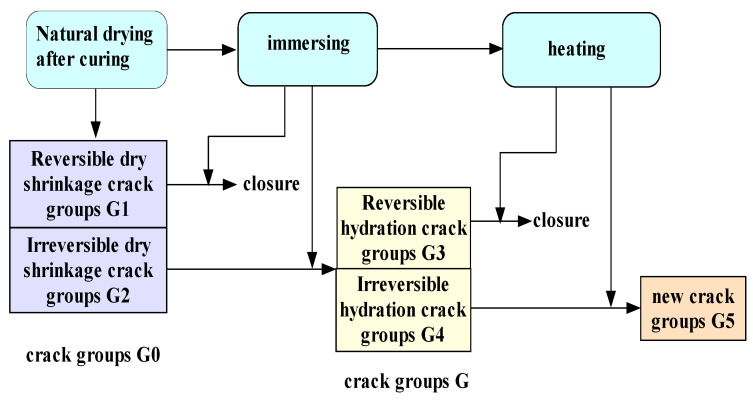
The evolution process of microcrack in concrete [27].

The influence of high temperature on the elastic modulus of concrete is not only related to the aggregate, water/cement ratio, size and shape of the specimen but also depends on the hydration degree and moisture content of the matrix. After curing and natural drying, liquid water (including capillary water, physical adsorption water, interlayer water, etc.) is stored inside the concrete specimen. At the same time, some initial microcracks are derived. In particular, many dry shrinkage microcracks are generated during the natural drying process, including reversible and irreversible microcracks [29]. These dry shrinkage microcracks are more frequent in the interfacial transition zone between aggregate and cement mortar. When the natural drying concrete absorbs water again, the air in the microcracks is replaced by water penetrating the specimen. The water at the crack tip has a tension that promotes crack propagation. Therefore, after naturally drying concrete is soaked in water, the reversible crack closes, and the irreversible crack will constantly derive, grow, and connect based on the original damaged nucleus due to the tension of water, developing new cracks, namely, hydration cracks [30,31,32]. At high temperatures, the water (including free water, physically adsorbed water, interlayer water and chemically bound water) in the wet concrete will vanish due to phase change, accompanied by a series of physical and chemical reactions. Under the combined humidity and heat conditions, the evolution process of microcracks in concrete can be referred to the research results of Li [27], as shown in Figure 8—the variation of concrete elastic modulus results from microcracks evolution under the coupling action of temperature and water.

(1) At room temperature, concrete elastic modulus has water strengthening effect, that is, the humidity-heat coupling factor DIF (T = 20 °C, W) ≥ 1, for the following reasons:
  ① When the natural drying concrete is soaked in water, the unhydrated cement can be rehydrated or the exudated calcium hydroxide is carbonized to form calcium carbonate, which has a refining effect on the microporous structure of concrete [33]. In addition, the dry shrinkage reversible cracks G1 formed in the natural drying process of concrete may heal when they are exposed to water. Both the refinement of the microporous structure and the closure of reversible cracks will increase the elastic modulus.
  ② As the moisture content of concrete rises, the pores and cracks inside the concrete are filled with free water and physically adsorbed water. The compressibility of water is far less than that of air, and the viscosity of water is greater than that of air. The water in the microcrack is subjected to great resistance when flowing under the load. Moreover, as a part of the concrete, the water can also bear a certain load, making the concrete rigidity increase. However, the water accumulated at the crack tip has a tension that promotes the irreversible cracks G2 to expand and form new hydration G from the original damaged nucleus. The pore water pressure inside the cracks at T = 20 °C is small, and the splitting effect of water is weak. Compared with the healing of reversible dry shrinkage cracks and the strengthening effect of water replacing air on the elastic modulus, the evolution of hydration crack at room temperature has a less weakening effect on the elastic modulus. Therefore, the elastic modulus of wet concrete at room temperature is larger than dry concrete.
(2) At high temperatures, the elastic modulus of concrete under different moisture contents declines with the rise of temperature T. During heating, the liquid water inside the wet concrete is gradually replaced by vapor. As mentioned above, the compressibility of vapor is larger than that of water, while the viscosity of vapor is smaller than that of water, which reduces the elastic modulus of concrete. In addition, the irreversible dry shrinkage cracks formed during the curing and drying of concrete specimens and the irreversible hydration cracks formed during secondary hydration will further evolve with the increase of internal pore pressure and the occurrence of physicochemical reaction, resulting in the decline of concrete elastic modulus. The decline of concrete elastic modulus caused by water phase change and microcrack propagation due to temperature rise is the temperature-weakening effect of elastic modulus. Under the humidity-heat condition, the variation of elastic modulus of concrete results from the competition between the strengthening effect of water and the weakening effect of temperature, and the DIF in different temperature ranges show different characteristics.
  ① When the ambient temperature T of concrete with different moisture content is less than its characteristic temperature T_w_, that is, T < T_w_. However, DIF decreases with increasing temperature. There is still DIF (T, W) ≥ 1. Within this temperature range, free water and physically adsorbed water inside concrete materials gradually lose, and the phase change of water absorbs a lot of heat. The hydration cracks G formed during concrete immersion can be divided into reversible cracks G3 and irreversible cracks G4. Cracks G3 heal with water loss, and cracks G4 evolve further with the temperature rise. At this stage, the crack growth is relatively stable, and the growth rate is relatively slow. Compared with the strengthening effect of the water kept in micro cracks and closure of the cracks G3, the weakening effect of the irreversible cracks G4 growth caused by temperature and phase change on the elastic modulus is weak. Therefore, within this temperature range, the elastic modulus of wet concrete is larger than that of stoved concrete at T = 20 °C.
  ② When ambient temperature T is greater than characteristic temperature T_w_ and less than critical temperature T_c_, that is, T_w_ < T < T_c_, then DIF(T,W) < l, The critical temperature T_c_ is about 500–550 °C. With the rise of temperature and loss of liquid water, cracks grow further, and the crack growth rate increases with the rise of the moisture content of wet concrete. This is because the longer the soak time of concrete, the more irreversible hydration cracks will be. Although the elastic modulus of wet concrete at room temperature is larger than that of dry concrete due to the strengthening effect of water, with the rise of temperature, the internal interlayer water and chemically bound water are gradually lost, and the internal energy is transformed into the surface energy required for cracks propagation. Therefore, the higher the initial moisture content before heating, the more cracks are generated during the water phase change process and the faster the rate of elastic modulus decline. However, at this stage, the weakening effect of temperature on the elastic modulus is still weakened by the water that has not yet been lost. Compared with the elastic modulus of the stoved concrete, the elastic modulus of humidity-heat coupling is smaller than that at T = 20 °C, but larger than that at the same high temperature.
  ③ When the ambient temperature T is greater than the critical temperature T_c_, T > T_c_, then DIF(T,W) < 1, the liquid water inside the crack disappears completely with the end of phase change and is replaced by gas. Crack groups formed in the hydration process and physicochemical reaction process grow rapidly at high temperatures. Similar to the previous stage, the higher the moisture content, the more the number of derived cracks in the concrete after the water phase change, the faster the concrete damage evolution rate, the faster the elastic modulus decline rate, and the smaller the elastic modulus value. Therefore, the moisture content shows an “apparent weakening effect” on the elastic modulus of concrete in this temperature range, but in fact, it is not the water that has a weakening effect on the elastic modulus, but the cracks G5 derived from inside concrete after the water phase change and disappearance. The new cracks G5 expand rapidly with the increase of temperature until the main crack that leads to the fracture of the specimen is formed. In this temperature range, the elastic modulus of wet concrete is less than that of stoved concrete at the same temperature.

## 5. Conclusions

In this paper, the coupling effect of moisture content and temperature on the elastic modulus of concrete is experimentally investigated. Based on the experimental results, the mechanism of the humidity-heat coupling effect of elastic modulus was analyzed, and the following observations were made:

(1) Under the humidity-heat condition, the relationship between the variation factor DIF of elastic modulus and moisture content can be expressed as DIF(T,W) = A(T) + B(T)W, where the fitting coefficients A(T) and B(T) depend only on temperature. When T = 20 °C, DIF(T,W) = K_w_(T), A(T) and B(T) degenerate into constants h and g. The elastic modulus of concrete declines with the rise of temperature, and the higher the moisture content, the more significant the decline rate of elastic modulus.
(2) Under the humidity-heat condition, the equation of the variation factor DIF of elastic modulus with temperature can be expressed as: DIF = b/[1 + e^p(T*−u)^], where the fitting coefficients b, p and u are functions of moisture content. The high-temperature elastic modulus of natural drying concrete decreases with the increase in temperature. The relationship between the elastic modulus of natural drying concrete variation factor K_T_ and temperature can be expressed as K_T_ = a/[1 + e^−m(T*−n)^], where the fitting coefficients a, m and n are constants.
(3) Within a certain temperature range (T ≤ 375 °C, 375 °C is the characteristic temperature of saturated concrete), there is an equivalent relation between the influence of moisture content and temperature on elastic modulus: $W=b_{1}+b_{2}e^{-T_{w}^{*}/b_{3}}$. When T is less than the characteristic temperature T_w_, DIF > 1. The water-strengthening effect of the elastic modulus is greater than the temperature-weakening effect; When the temperature T is greater than the characteristic temperature T_w_, there is DIF < 1. The weakening effect of temperature is greater than the strengthening effect of water. If the moisture content is increased or the ambient temperature is lowered, the elastic modulus will increase; however, if the moisture content is decreased or the temperature is raised, the elastic modulus will decrease. In concrete structure engineering, changing the modulus of elastic modulus can be achieved by controlling the moisture content and temperature of the concrete according to the equivalent relation of the two.
(4) As the moisture content increases, the variation of the elastic modulus shows different trends with the critical temperature T_c_ (the critical temperature T_c_ is about 500–550 °C) as the boundary. When the temperature T < T_c_, the water stored in concrete before heating presents a strengthening effect on the elastic modulus; When the temperature T > T_c_, the water stored in concrete before heating on the elastic modulus presents an “apparent weakening effect”. In this case, the decrease of the elastic modulus is caused by the deterioration of the internally derived microcracks after the water disappears.
(5) The variation of the elastic modulus of concrete results from the phase change of water and the evolution of cracks under the coupled action of heat and humidity. When the strengthening effect of water and reversible crack healing on the elastic modulus is dominant, the elastic modulus of concrete material increases; conversely, when the weakening effect of crack propagation on the elastic modulus is dominant, the elastic modulus of concrete material decreases. Based on our existing research results and macroscopic experiments, the variation pattern of elastic modulus under the coupling condition of heat and humidity is explained by the evolutionary mechanism of cracks. However, micro-scale experiments need to be carried out for verification. In addition, the above research results are based on the heat-humidity coupling experiment of concrete with the same aggregate type and water/cement ratio (see Table 1). Further studies are needed to investigate the differences in the elastic modulus of concrete under the combined heat and humidity conditions that may be caused by different aggregate types and water-cement ratios.

## Figures and Tables

**Table 1:** Mix proportions of concrete specimen.

Cement (kg/m^3^)	Sand (kg/m^3^)	Aggregate (kg/m^3^)	Water (kg/m^3^)	Superplasticizer (kg/m^3^)
425	600	1132	184	8

**Table 2:** Fitted parameter values.

T/°C	A	B
20	1.00	0.08
200	0.82	0.07
400	0.77	0.04
520	0.68	0.01
620	0.58	−0.03

**Table 3:** The fitting coefficient value.

W	b	p	u
0	1.73	0.03	10.86
1.81	1.16	0.10	28.92
2.60	1.15	0.14	28.56
3.40	1.22	0.15	27.19
3.74	1.27	0.17	26.16

**Table 4:** Reference values of characteristic temperatures corresponding to different moisture content.

W/%	T_w_/°C
0	20
1.81	200
2.60	320
3.40	340
3.74 (saturation)	375

## Data Availability

Not applicable.
